# Phosphorus-solubilizing fungi promote the growth of *Fritillaria taipaiensis* P. Y. Li by regulating physiological and biochemical reactions and protecting enzyme system–related gene expression

**DOI:** 10.3389/fgene.2024.1459191

**Published:** 2025-01-07

**Authors:** Yueheng Wang, Lin Yuan, Yuhan Wang, Jiaqi Lang, Mingyan Ye, Qingqiu Liu, Qiang Ma, Nong Zhou

**Affiliations:** ^1^ Chongqing Engineering Laboratory of Green Planting and Deep Processing of Famous-Region Drug in the Three Gorges Reservoir Region, College of Biology and Food Engineering, Chongqing Three Gorges University, Chongqing, China; ^2^ Food and Drug Research Institute of Wanzhou, Chongqing, China; ^3^ Chongqing Key Laboratory of Development and Utilization of Genuine Medicinal Materials in Three Gorges Reservoir Area, Chongqing Three Gorges Medical College, Chongqing, China

**Keywords:** *Fritillaria taipaiensis* P. Y. Li, antioxidant oxidase, gene expression, phosphorus-solubilizing fungi, plant physiology and chemical reactions

## Abstract

**Introduction:**

*Fritillaria taipaiensis* P. Y. Li is a plant used to treat respiratory diseases such as pneumonia, bronchitis, and influenza. Its wild resources have become increasingly scarce, and the demand for efficient artificial cultivation has increased significantly in recent years. Phosphorus-solubilizing fungi can promote the dissolution of insoluble phosphate complex, which benefits plant nutrition. Another strategy for efficiently cultivating traditional Chinese medicine plants is to combine the soil with phosphorus-solubilizing fungi to provide nutrients and other desired features. This study aimed to investigate the effects of different phosphorus-solubilizing fungi and their combinations on photosynthesis, physiological and biochemical characteristics, and expression of protective enzyme system–related genes, and to find a reference strain suitable for the artificial cultivation and industrial development of *F. taipaiensis* P. Y. Li. In this study, the phosphorus-solubilizing fungi isolated from the rhizosphere soil of *F. taipaiensis* P. Y. Li were applied to the cultivation of *F. taipaiensis* P. Y. Li for the first time.

**Methods:**

In this study, seven treatment groups (S1-S7) and one control group were set up using indoor pots as follows: S1 (inoculation with *Aspergillus tubingensis*), S2 (inoculation with *A. niger*), S3 (inoculation with *Aspergillus nigerfunigatus*) and S4 (inoculation with *A. tubingensis* and *A. niger*), S5 (inoculation with *A. tubingensis* and *A. nigerfunigatus*), S6 (inoculation with *A. niger* and *A. nigerfunigatus*), S7 (inoculation with *A. tubingensis*, *A. niger*, and *A. nigerfunigatus*), and CK (control group). These strains were inoculated into pots containing *F. taipaiensis* P. Y. Li bulbs,and the effects of different phosphorus-solubilizing fungi and combinations on the photosynthetic characteristics, basic physiological and biochemical indicators, and differential gene expression of protective enzyme systems in *F. taipaiensis* P. Y. Li leaves were determined.

**Results:**

Most growth indexes showed significant differences in the fungal treatment groups compared with the CK group (*P* < 0.05). The stem diameter and plant height in the S5 group were the highest, which were 58.23% and 62.49% higher than those in the CK group, respectively. The leaf area in the S7 group was the largest, which increased by 141.34% compared with that in the CK group. Except for intercellular CO_2_ concentration (Ci), the contents of photosynthetic pigments, photosynthetic parameters, and amounts of osmoregulatory substances increased to varying degrees in the fungal treatment groups (*P* < 0.05). Among these, the S5 group had the highest stomatal conductance index and soluble sugar and free proline contents, whereas S6 had the highest chlorophyll a and soluble protein contents. In addition, the malondialdehyde (MDA) content in all inoculation groups was lower than that in the CK group. The MDA content was the lowest in S7, about 44.83% of that in the CK group. The activities of peroxidase (POD), superoxide dismutase (SOD), and catalase (CAT) were higher in all inoculation groups than those in the CK group; the changes in SOD and CAT activities were significant (*P* < 0.05). The expression levels of *FtSOD*, *FtPOD*, and *FtCAT* in the S5 group were the highest, which were 8.67, 7.65, and 6.08 times of those in the CK group, respectively.

**Conclusion:**

Various combinations of phosphorus-solubilizing fungi exhibit differential capacities to enhance plant growth indices (including leaf area, plant height, and stem diameter), promote the accumulation of photosynthetic pigments, regulate osmotic pressure, and elevate antioxidant activity. Notably, The three fungal combinations (S7) were prone to cause a certain degree of antagonism, leading to suboptimal performances in certain biochemical indicators, such as free proline and POD levels. Our study pointed out that the S5 group inoculated with *A. tubingensis* and *A. niger* had the best overall effect. These experimental results provided a theoretical basis for the selection and development of artificial cultivation of *F. taipaiensis* P. Y. Li.

## 1 Introduction

*Fritillaria taipaiensis* P. Y. Li is a perennial herb belonging to the lily family. It has a bitter taste and a slightly cold nature. It is a traditional Chinese medicinal material used to clear heat and nourish the lungs, relieve cough and phlegm, as well as reduce swelling and discharge pus (Xu et al., 2019). Wild *Fritillaria* resources have become increasingly scarce with a recent increase in market demand *F. taipaiensis* P. Y. Li is a new species of *Fritillaria* (Qu et al., 2022), and hence its artificial cultivation is also the most successful at present. This plant has become a well-known medicinal material that meets the market demand for *Fritillaria*. The artificial cultivation technology has become mature after 40 years of development (Lang et al., 2024). Although the altitude range suitable for planting has remarkably expanded after the successful introduction of *F. taipaiensis* P. Y. Li, the cultivation environment remains limited (Liu et al., 2022). It is worth noting that the growth and development, nutrient accumulation, and medicinal quality of *F. taipaiensis* P. Y. Li are influenced by various soil elements and conditions, including potential phosphorus deficiency. Therefore, in-depth research is research is required to improve yield and quality, thereby satisfying the ever-growing demand for traditional Chinese medicine and high-quality *F. taipaiensis* P. Y. Li.

Phosphorus is an element essential for plant growth. It is involved in synthesizing many crucial organic compounds, including enzymes and coenzymes, and can promote photosynthesis and accelerate root development (Lambers et al., 2022). However, plants can absorb less than 3% of the soil’s phosphorus, and almost half of the arable land in China is deficient in phosphorus. The phosphorus fertilizers can only increase the amount of plant-absorbed phosphorus to some extent because phosphorus easily forms phosphates, which are difficult to dissolve in the soil. Hence, it is an ineffective form of phosphorus that plants cannot easily absorb (Ameen et al., 2019; Silva et al., 2023). This may also lead to soil erosion, soil compaction, and other problems, thereby severely damaging the ecological environment (Ye, 2024). Phosphorus-solubilizing fungi can convert soluble or insoluble phosphate in soil into organic phosphorus compounds, which plants easily absorb. These fungi reduce the soil pH by producing acetic acid, lactic acid, malic acid, and other organic acids, so that phosphate can be dissolved under acidic conditions and soil phosphorus can be activated. Therefore, the application of phosphorus-solubilizing fungi has become a critical method of overcoming the shortage of phosphorus in plants or its incomplete absorption by plants. When applied, these fungi can promote plant growth and development and accelerate the production of beneficial active substances in plants. Nowadays, the influence of rhizosphere microorganisms on plant growth is frequently explored (Saeed et al., 2021; Yuan et al., 2022; Wu et al., 2024). Investigating rhizosphere microorganisms is also markedly significant for biofertilizer development because it improves the soil environment and reduces chemical fertilizer-induced pollution (Niu et al., 2021; Zhang et al., 2024). At present, phosphorus-solubilizing bacteria are more widely used in crop production than phosphorus-solubilizing fungi. Ceci et al. (2018) reported that many bacteria lose their phosphorus-solubilizing ability during their isolation and purification, but fungi maintain their phosphorus-solubilizing effect. However, this phosphorus-enhancing mechanism of fungi and the interaction between beneficial fungi isolated from *F. taipaiensis* P. Y. Li rhizosphere soil and the plant itself have remained largely unexplored.

In this study, the effect of phosphorus-solubilizing fungi on the growth of *F. taipaiensis* P. Y. Li was explored. Different phosphorus-solubilizing fungal strains or their combinations were applied to *F. taipaiensis* P. Y. Li. The contents of photosynthetic pigments, various protective enzymes, polysaccharides, and proteins were considered indicators of the plant growth status. Therefore, we explored the effects of the applied fungi on the growth and development, photosynthesis, and antioxidant activities of *F. taipaiensis* P. Y. Li. The optimal strains or flora were selected through comparative analysis, which served as an effective reference value for the cultivation technology of *F. taipaiensis* P. Y. Li and biofertilizer development.

## 2 Materials and methods

### 2.1 Test materials

The bulbils of *F*. *taipaiensis* F. Y. Li used in this experiment were provided by the *F. taipaiensis* F. Y. Li Planting Base located in Hongchiba, Wuxi County, Chongqing Municipality (N31°36′26.07″, E108°49′18.75″), and were identified as four-year-old bulbils of *F*. *taipaiensis* F. Y. Li (belonging to the Liliaceae family) by Professor Zhou Nong of Chongqing Three Gorges University. These bulbils underwent a rigorous screening process to ensure uniformity in size and quality. The cultivation experiments were conducted between 2022 and 2023 at the *F*. *taipaiensis* F. Y. Li Planting Base within the Hongchiba Scenic Area. The test strains of phosphorus-solubilizing fungi used in this study were ESA3 (*Aspergillus tubingensis*), GYB1 (*Aspergillus niger*), and TBXB2 (*Aspergillus nigerfunigatus*). These strains were rigorously screened from 29 strains of phosphorus-solubilizing fungi isolated from the rhizosphere soil of *F*. *taipaiensis* P. Y. Li collected from ten different locations in China. The screening process first involved the use of the phosphorus-solubilizing ring method for initial selection, followed by the molybdenum-antimony resistance colorimetric method to further identify highly efficient phosphorus-solubilizing fungi. Ultimately, the three dominant strains were determined based on colony morphology and the amplified ITS sequence results. The test soil comprising yellow loam, river sand, and organic fertilizer (2:1:1) was obtained from the Chongqing Three Gorges University. The soil was sieved with a 2 mm sieve, sterilized at 121°C for 2 h, and left for 7 days before use.

### 2.2 Experimental design

The study was conducted in October 2022 at the Hongchiba *F. taipaiensis* P. Y. Li Planting Base in Wuxi County, Chongqing Municipality. The plants were potted in plastic flowerpots (diameter: 15 cm; height: 18 cm), which were wiped three times with 75% ethanol solution before planting. Seven treatment groups, namely S1, S2, S3, S4, S5, S6, and S7, and a control group (CK) were established in the experiment. The soil in the CK group was subjected to high-temperature sterilization without any fungal treatment. Ten replicates were established in each treatment group, and approximately 5 *F. taipaiensis* P. Y. Li plants were planted in each pot. The plants were placed in the same area to ensure that all the test samples were exposed to the same elevation, light, temperature, and humidity. In order to ensure the robustness and statistical significance of the experimental results, the fungal treatment solutions were applied twice in March and April of 2023, respectively. Each pot received 100 mL of fungal suspension of concentration 2 × 10^8^ CFU/mL, whereas the control group received an equal amount of sterile water. During planting, the plants were maintained according to the conventional management method used for *F. taipaiensis* P. Y. Li. The inoculation methods were single plant, mixed two plants, and mixed three plants. A random grouping design was used to allocate plants to different inoculation methods, ensuring that any environmental or other confounding factors were evenly distributed between groups. Each treatment group had 10 biological replicates. Table 1 presents the specific inoculation methods.

**TABLE 1:** Inoculated strains and inoculated amounts in different treatment groups.

Group	Inoculated strain	Inoculation measurement
S1	*Aspergillus tubingensis*	100 mL/strain
S2	*Aspergillus niger*	100 mL/strain
S3	*Aspergillus nigerfunigatus*	100 mL/strain
S4	*Aspergillus tubingensis*, *Aspergillus niger*	50 mL/strain
S5	*Aspergillus tubingensis*, *Aspergillus nigerfunigatus*	50 mL/strain
S6	*Aspergillus niger*, *Aspergillus nigerfunigatus*	50 mL/strain
S7	*Aspergillus tubingensis*, *Aspergillus niger*, *Aspergillus nigerfunigatus*	33 mL/strain
CK	without adding fungal	100 mL sterile water

### 2.3 Index measurement

#### 2.3.1 Measurement of growth indexes and photosynthetic parameters

To gather experimental data, in June 2023 during sunny weather, we randomly selected six plants from each parallel group and measured the growth index for each plant pot. The plant height was measured using a tape measure, and the plant height from its top to the soil matrix interface was used as the measurement standard. The root was measured using a tape measure, and the length from the root base to the longest root was established as the measurement standard. A leaf from each plant was randomly selected for measurement. The leaf area was measured using a portable laser leaf area scanner (CI-203, CID Bio-Science, Inc.) after completely unfolding the leaf. Four photosynthetic parameters, namely net photosynthetic rate (Pn), stomatal conductance (Gs), intercellular CO_2_ concentration (Ci), and transpiration rate (Tr), were evaluated using a photosynthesis analyzer (LI-6400, LI-COR, Inc., United States) between 11 a.m. and 1 p.m. when the stomatal opening of the leaves was the greatest, following the method outlined by Wu et al. (2021).

#### 2.3.2 Determination of the contents of leaf photosynthetic pigments

In this experiment, six potted plants under sunny weather conditions were selected from each parallel group, and the growth index of one plant in each pot was measured. We applied the method proposed by Croft et al. (2017) to extract the chlorophyll from *F. taipaiensis* P. Y. Li leaves using a mixture containing certain proportions of acetone, water, and anhydrous ethanol. The absorbance value at a specified wavelength was determined using an ultraviolet spectrophotometer (UV-2450, Shimadzu Group, Japan). Also, chlorophyll a, chlorophyll b, and carotenoid contents were calculated.

#### 2.3.3 Measurement of physiological and biochemical indexes

In this experiment, six potted plants under sunny weather conditions were selected from each parallel group, and the growth index of one plant in each pot was measured. The enzymatic activities within the leaf’s protective enzyme system were determined following the methodology described by Hayashi and Palmgren (2021). The peroxidase (POD) activity was measured using the guaiacol method, where an increase of 0.01 in absorbance at 470 nm per minute was considered as one unit of enzyme activity. The catalase (CAT) activity was measured via ultraviolet (UV) spectrophotometry, defining a unit of enzyme activity as a 0.1 decrease in absorbance at 240 nm (A240 nm). The superoxide dismutase (SOD) activity was determined by the nitro blue tetrazolium (NBT) photochemical reduction method, defining one unit of enzyme activity as 50% inhibition of NBT photochemical reduction. Additionally, malondialdehyde (MDA) and soluble sugar contents were assessed using the thiobarbituric acid colorimetric method. The soluble protein content was determined using the Thomas Brilliant Blue method, and free proline content was quantified by UV spectrophotometry. The soluble protein content was determined using the Coomassie brilliant blue method (Shams et al., 2024). The free proline content was determined using the UV method. The SOD activity was determined using the nitrogen blue tetrazole photochemical reduction method (Fernández-Marín et al., 2018; Shams et al., 2023). The POD activity was determined using the guaiacol method. The CAT activity was determined through UV spectrophotometry.

#### 2.3.4 Differential analysis of protective enzyme system–related gene expression in *Fritillaria taipaiensis* P. Y. Li leaves

In this experiment, the TRIzol Plus RNA Purification Kit (Thermo Fisher Scientific, United States) and RNase-Free DNase Set (QIAGEN, Germany) reagents were used to isolate and purify RNA from *F. taipaiensis* P. Y. Li leaves through repeated high-speed freezing centrifugation. The RNA content, purity, and quality were determined through UV spectrophotometry and electrophoresis, with an optical density value (A260/A280) requirement of 1.8–2.1. Then, the RNA was reverse transcribed into first-strand cDNA using a reverse transcription kit (Thermo Fisher Scientific, United States), and the obtained cDNA was stored at −20°C for later use. Real-time quantitative polymerase chain reaction (RT-qPCR) was performed on a real-time fluorescence quantitative instrument (CFX96, Bio-Rad, United States) using Taq Pro Universal SYBR qPCR Master Mix (Novogene, Wuhan, China) following the manufacturer’s protocols. The primer sequences designed for the highly specific fragment of the target genes, which could avoid the nonspecific amplification, are listed in Table 2. The fluorescence quantitative reaction system comprised 10 µL of 2 × Taq Pro Universal SYBR qPCR Master Mix, 1 μL of cDNA template, 0.4 μL each of forward and reverse primers, and an appropriate amount of water, with a total volume of 20 μL. The reaction conditions were as follows: 95°C for 2 min, 95°C for 20 s, and 58°C for 20 s, for 39 cycles. A melting curve was also plotted, and three biological replicates were set. Finally, the relative gene expression levels were calculated using the 2^−△△Ct^ method using *rpl16* as the reference gene (Adnan et al., 2011).

**TABLE 2:** Real-time PCR primers and conditions.

Gene name	Primer name	Primer sequence	Product length (bp)
*FtSOD*	F	TTC​AGT​TTC​TTA​GTG​ACA​ATA​GGC​G	195
	R	GGT​CTT​AGT​CTG​GAT​ACG​GCA​A	
*FtPOD*	F	TTT​CCT​TTC​CAT​TCA​CCC​G	175
	R	AAG​ACC​CTT​CCC​TTT​GTT​CG	
*FtCAT*	F	TAT​TCC​ACA​ACA​ACG​AAA​GCA​C	183
	R	GGA​CCC​GAA​TCC​GTT​AGT​ATG	
*rpl16*	F	TTC​GTG​CTA​CAT​TCG​TAG​GGT​C	190
	R	GTT​CCA​TTG​CGG​AGT​TCG​G	

Note: *rpl16* is reference gene.

### 2.4 Data processing

All data and statistical analyses were performed using Microsoft Excel (Microsoft Office Professional 2021; version 2,407 Build 16.0.17830.20166) and SPSS statistical software (IBM SPSS Statistics for Windows, version 23.0). The Origin 2023 software package (OriginLab Corporation, United States) was used for plotting and visualization.

## 3 Results

### 3.1 Effects of phosphorus-solubilizing fungi on the growth index of *Fritillaria taipaiensis* P. Y. Li

The growth index of *F. taipaiensis* P. Y. Li inoculated with different phosphorus-solubilizing fungi is shown in Table 3. The growth index in almost all treatment groups was significantly higher than that in the CK group. The stem thickness and plant height in the S5 group were the highest, which were 58.23% and 62.49% higher than those in the CK group, respectively. The two growth indexes in the S1, S2, and S6 groups were at the same level, but lower than those in the S5 group. The leaf thickness in the S1 group was the highest, 17.62% higher than that in the CK group. The leaf thickness in the S2 group was at the same level as that in the CK group. The leaf area in the S7 group was the largest, which was 141.34% higher than that in the CK group. The leaf area in the S5 group was 136.71% higher than that in the CK group. No significant difference was found between the S7 group and the S7 group. The leaf area in the S1 group was the smallest, which was only 44.49% higher than that in the CK group.

**TABLE 3:** Growth indexes of *Fritillaria taipaiensis* P. Y. Li plants under different treatments (±sd, *n* = 10).

Group	Stem thickness (mm)	Leaf thickness (mm)	Plant height (cm)	Leaf area (cm^2^)
CK	0.924 ± 0.0358d	0.772 ± 0.0376b	5.116 ± 0.1916d	3.367 ± 0.5403e
S1	1.404 ± 0.0940b	0.908 ± 0.0476a	7.260 ± 0.2600b	4.865 ± 0.6739d
S2	1.264 ± 0.0847b	0.787 ± 0.0350b	7.453 ± 0.2155b	5.787 ± 1.2507cd
S3	1.332 ± 0.0683c	0.832 ± 0.0701ab	6.317 ± 0.1767c	6.339 ± 1.2048bc
S4	1.194 ± 0.0114c	0.812 ± 0.0630b	6.33 ± 0.1758c	6.369 ± 0.6302bc
S5	1.462 ± 0.0792a	0.822 ± 0.0884ab	8.313 ± 0.1793a	7.970 ± 0.6279a
S6	1.204 ± 0.0434b	0.848 ± 0.0653 ab	7.340 ± 0.1637b	7.484 ± 0.7596 ab
S7	1.068 ± 0.0303c	0.856 ± 0.0677ab	6.363 ± 0.1601c	8.126 ± 0.8995a

Note: The data are expressed as means ± standard deviation. Different letters in the same column indicate significant differences at *P* < 0.05 levels, as detected using LSD test.

### 3.2 Effects of phosphorus-solubilizing fungi on leaf area and photosynthetic parameters of *Fritillaria taipaiensis* P. Y. Li

Table 4 presents the effects of the inoculation of different phosphorus-solubilizing fungi on the leaf area and photosynthetic parameters of *F. taipaiensis* P. Y. Li. The leaf area in the treatment groups was significantly higher than that in the CK group (*P* < 0.05). The leaf area of *F. taipaiensis* P. Y. Li inoculated with different fungal strains exhibited significant changes. The average middle lobe area in the S7 group inoculated with a mixture of the three strains was the highest, 155.53% higher than that in the CK group. In contrast, the leaf area in the S1 group was the smallest and was only 52.99% higher than that in the CK group. Inoculation with different phosphorus-solubilizing fungi exerted significant effects on plant photosynthesis in the same habitat.

**TABLE 4:** Leaf area and photosynthetic parameters index measurement of *Fritillaria taipaiensis* P. Y. Li (±sd, *n* = 10).

Group	Leaf area (cm^2^)	Pn (μmol·m^−2^·s^−1^)	Gs (μmol·m^−2^·s^−1^)	Ci (μmol·m^−2^·s^−1^)	Tr (mmol·m^−2^·s^−1^)
CK	3.180 ± 0.530e	5.695 ± 0.013d	0.046 ± 0.002e	162.25 ± 1.708bcd	0.547 ± 0.062d
S1	4.865 ± 0.670d	11.950 ± 2.195bc	0.091 ± 0.021c	155.50 ± 0.577d	1.750 ± 0.352b
S2	5.787 ± 1.250cd	12.500 ± 0.082b	0.096 ± 0.001bc	161.50 ± 1.291cd	1.830 ± 0.008b
S3	6.339 ± 1.200bc	10.950 ± 0.129c	0.069 ± 0.001d	120.50 ± 1.291e	1.355 ± 0.013c
S4	6.369 ± 0.630bc	12.450 ± 0.129b	0.084 ± 0.006c	171.75 ± 0.957b	2.075 ± 0.013a
S5	7.970 ± 0.630a	10.950 ± 0.695c	0.101 ± 0.001b	189.50 ± 1.291a	1.620 ± 0.064b
S6	7.484 ± 0.760ab	11.350 ± 0.580bc	0.090 ± 0.014c	149.00 ± 1.414bcd	1.720 ± 0.248b
S7	8.126 ± 0.900a	14.075 ± 0.096a	0.121 ± 0.001a	171.50 ± 1.291bc	2.272 ± 0.001a

Note: Pn is the net photosynthetic rate, Gs is the stomatal conductance, Ci is the intercellular CO_2_ concentration, and Tr is the transpiration rate. The data are expressed as means ± standard deviation. Different letters in the same column indicate significant differences at *P* < 0.05 levels, as detected using LSD test.

Except for Ci, the average values of the other parameters were higher in the treatment groups than in the CK group. The Pn and Tr in the S7 group were the highest. They were 147.15% and 315.36% higher in the S7 group than in the CK group, respectively. The increase in Gs in the S5 group was the highest, 119.57% higher than that in the CK group. Other than the three single fungus groups S1, S2, and S3, all other mixed fungi groups displayed improved Ci value of the *F. taipaiensis* P. Y. Li leaves. The most obvious improvement was noted in the S5 group, which was 16.8% higher than that in the CK group. All groups displayed significant differences.

### 3.3 Effects of inoculation with different phosphorus-solubilizing fungi on photosynthetic pigment content in leaves

As shown in Table 5, the photosynthetic pigment content of leaves in some treatment groups was significantly higher than that in the CK group. The chlorophyll content was the highest in the S6 group, followed by the S3 and S7 groups. The chlorophyll content in the S6 group was 16.8% higher than that in the CK group. The chlorophyll b content in the S3 group was the highest, followed by the S6 group. It was 29.4% and 14.7% higher in the S3 and S6 groups, respectively, than in the CK group. The carotenoid content increased most significantly in the S1 group, followed by the S3 and S2 groups. The single fungus treatment groups exhibited a greater increase in the carotenoid content compared with the combined fungi treatment groups.

**TABLE 5:** Determination of photosynthetic pigment content in *Fritillaria taipaiensis* P. Y. Li (±sd, *n* = 10).

Group	Chlorophyll a (mg·g^−1^)	Chlorophyll b (mg·g^−1^)	Carotenoids (mg·g^−1^)	Chlorophyll a/b
CK	0.808 ± 0.002c	0.265 ± 0.007c	0.215 ± 0.011a	3.049 ± 0.080c
S1	0.851 ± 0.027bc	0.291 ± 0.014b	0.274 ± 0.007a	2.924 ± 0.061 ab
S2	0.856 ± 0.006b	0.285 ± 0.002bc	0.249 ± 0.002a	3.004 ± 0.002 ab
S3	0.921 ± 0.008a	0.343 ± 0.013a	0.258 ± 0.002a	2.685 ± 0.358a
S4	0.844 ± 0.024bc	0.291 ± 0.013b	0.247 ± 0.012a	2.900 ± 0.049b
S5	0.852 ± 0.049bc	0.291 ± 0.010b	0.233 ± 0.010a	2.928 ± 0.067 ab
S6	0.944 ± 0.025a	0.304 ± 0.029b	0.241 ± 0.003a	3.105 ± 0.074 ab
S7	0.910 ± 0.047a	0.300 ± 0.008b	0.246 ± 0.017a	3.033 ± 0.080 ab

Note: The indicators in the table were measured using fresh leaves. The unit “g” represents the weight of the leaves. The data are expressed as means ± standard deviation. Different letters in the same column indicate significant differences at *P* < 0.05 levels, as detected using LSD test.

### 3.4 Effects of phosphorus-solubilizing fungi on MDA, soluble sugar, soluble protein, and free proline contents in *Fritillaria taipaiensis* P. Y. Li

The inoculation with phosphorus-solubilizing fungi significantly affected the MDA content in *F. taipaiensis* P. Y. Li (*P* < 0.05). The MDA content in all treatment groups was lower than that in the CK group. The content exhibited the largest reduction in the S7 group, followed by the S5 group. It was 44.83% and 37.93% lower in the S7 and S5 groups than in the CK group, respectively (Table 6).

**TABLE 6:** Determination of MDA, soluble sugar, soluble protein and free proline in the leaves of *Fritillaria taipaiensis* P. Y. Li (±sd, *n* = 10).

Group	MDA content (μmol·g^−1^)	Soluble sugar (μmol·g^−1^)	Soluble protein (μmol·g^−1^)	Free proline (μg·g^−1^)
CK	0.029 ± 0.0002a	0.219 ± 0.0053f	23.963 ± 0.4223e	104.503 ± 1.8075e
S1	0.025 ± 0.0040b	0.313 ± 0.0097d	23.986 ± 1.2156e	157.341 ± 1.4629a
S2	0.021 ± 0.0004bc	0.244 ± 0.0080e	37.580 ± 0.6294c	114.342 ± 0.8999d
S3	0.021 ± 0.0008bc	0.334 ± 0.0053c	39.518 ± 0.6643bc	149.284 ± 1.4316b
S4	0.019 ± 0.0006cd	0.357 ± 0.0095c	41.433 ± 1.5476b	134.338 ± 0.4268c
S5	0.018 ± 0.0010cd	0.599 ± 0.0049a	47.242 ± 1.2073a	157.341 ± 1.4629a
S6	0.019 ± 0.0010cd	0.402 ± 0.0082b	48.618 ± 1.0574a	149.284 ± 1.4316b
S7	0.016 ± 0.0007d	0.352 ± 0.0055c	31.564 ± 0.7069d	80.585 ± 0.6482f

Note: The data are expressed as means ± standard deviation. Different letters in the same column indicate significant differences at *P* < 0.05 levels, as detected using LSD test.

The inoculation with phosphorus-solubilizing fungi also significantly improved the soluble sugar and protein contents in *F. taipaiensis* P. Y. Li (Table 5). All treatment groups exhibited an increase in these contents compared with the CK group. The soluble sugar content was 173.52% in the S5 group compared with the CK group. The increase in the soluble protein content was the largest in the S6 group, followed by the S5 group. This increase in the S6 group was 102.89% higher than that in the CK group. The weakest improvement effect was noted in the S1 and S2 groups. The soluble protein content in the S1 group was almost the same as that in the CK group.

The inoculation with different phosphorus-solubilizing fungi significantly affected the free proline content in *F. taipaiensis* P. Y. Li (*P* < 0.05). The free proline content in the treatment groups, except for the S7 group, was higher than that in the CK group. The S5 group had the most obvious improvement effect. The proline content in the S5 group was 52.84% higher than that in the CK group, followed by the S6 and S4 groups. The proline content increased by 42.85% and 28.55%, respectively, in the S6 and S4 groups compared with the CK group. The free proline content was 22.89% lower in the S7 group than in the CK group. This was speculated to be caused by the antagonism between *A. niger* and the other two fungi, which led to a decrease in the free proline content rather than an increase. Based on the aforementioned findings on soluble sugar and protein contents, we could reasonably infer that the effects of the three phosphorus-solubilizing fungi used individually were lower than those of the combined strains. This was because when the fungi worked together, it led to antagonism.

### 3.5 Effects of phosphorus-solubilizing fungi on the protective enzyme activity of *Fritillaria taipaiensis* P. Y. Li

Table 7 shows the effects of the inoculation with phosphorus-solubilizing fungi on the activities of various protective enzymes of *F. taipaiensis* P. Y. Li. The protective enzyme activity values were higher in all treatment groups compared with the CK group. The enzyme activity was higher in most combined fungi treatment groups than in the single fungus treatment group. On analyzing all the measured data, we noted that the SOD activity enhancement effect in the S5 group was the best, followed by the S3 group. This effect was approximately 159.06% and 136.53% higher in the S5 and S3 groups than in the CK group, respectively. The POD enhancement effect in the S3 group was the best, which was approximately 86.00% higher than that in the CK group, followed by the S5 and S6 groups, which exerted the same enhancement effect. The POD activity enhancement effect did not increase but decreased in the S7 group and was 56.98% lower than that in the CK group. The CAT activity enhancement effect in the S6 group was the most obvious, which was 228.48% higher than that in the CK group, followed by the S5 and S7 groups. The three different combinations of strains more significantly improved the protective enzyme activity. The S3 group exerted a better promoting effect than the other two groups of single fungus. Among these, the S7 group reduced the POD enzyme activity. Thus, we speculated that *A. niger* and *A. nigerfunigatus* had a certain degree of antagonism, which also explained why the enhancement effect on the SOD and POD enzyme activities was not as expected in the S6 group.

**TABLE 7:** Protective enzyme system of *Fritillaria taipaiensis* P. Y. Li (±sd, *n* = 10).

Group	SOD activity	POD activity	CAT activity
CK	213.861 ± 2.800h	187.427 ± 0.621c	154.435 ± 0.977f
S1	244.284 ± 1.410g	177.947 ± 0.999d	314.351 ± 0.442g
S2	377.321 ± 0.985e	158.783 ± 0.595e	218.312 ± 0.730b
S3	505.854 ± 1.389b	348.614 ± 0.837a	345.301 ± 0.993a
S4	444.352 ± 1.406c	143.917 ± 0.935f	338.662 ± 0.899d
S5	554.019 ± 1.402a	207.669 ± 0.763b	395.607 ± 0.912e
S6	360.487 ± 1.404f	208.099 ± 0.744d	507.283 ± 0.871c
S7	382.335 ± 1.404d	80.631 ± 0.621g	383.693 ± 0.977h

Note: The data are expressed as means ± standard deviation. Different letters in the same column indicate significant differences at *P* < 0.05 levels, as detected using LSD test.

### 3.6 RT-qPCR analysis of genes related to the protective enzyme system of *Fritillaria taipaiensis* P. Y. Li treated with phosphorus-solubilizing fungi

**FIGURE 1:**
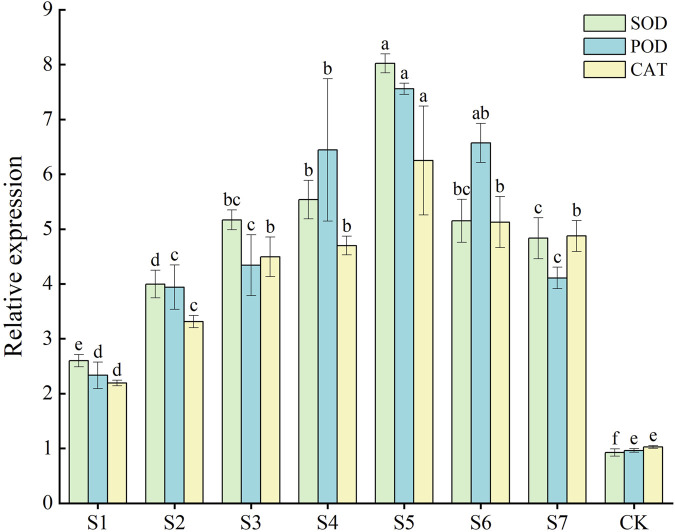
*FtSOD*, *FtPOD*, and *FtCAT* genes related to the protective enzyme system in leaves of *Fritillaria taipaiensis* P. Y. Li.

RT-qPCR was performed to determine the gene expression of three crucial protective enzymes in *F. taipaiensis* P. Y. Li. The gene expression levels of *FtSOD*, *FtPOD*, and *FtCAT* increased in all treatment groups compared with the control group. The gene expression of the three enzymes was higher in most combined strain groups than in the single-strain treatment groups. The *FtSOD*, *FtPOD*, and *FtCAT* activities increased 8.67, 7.65, and 6.08 times in the S5 group compared with the CK group, respectively. As shown in Figure 1, the effect on the gene expression levels was the best in the S5 group, followed by the combination of the S4 and S6 groups. *FtPOD* and *FtCAT* gene expression was higher, but the *FtSOD* gene expression was lower, in the S6 group than in the S4 group. The S7 group exhibited the lowest expression of genes related to the three enzymes. The *FtSOD* and *FtPOD* expression was lower in the S7 group than in the S3 single-strain treatment group.

### 3.7 Correlation analysis

**FIGURE 2:**
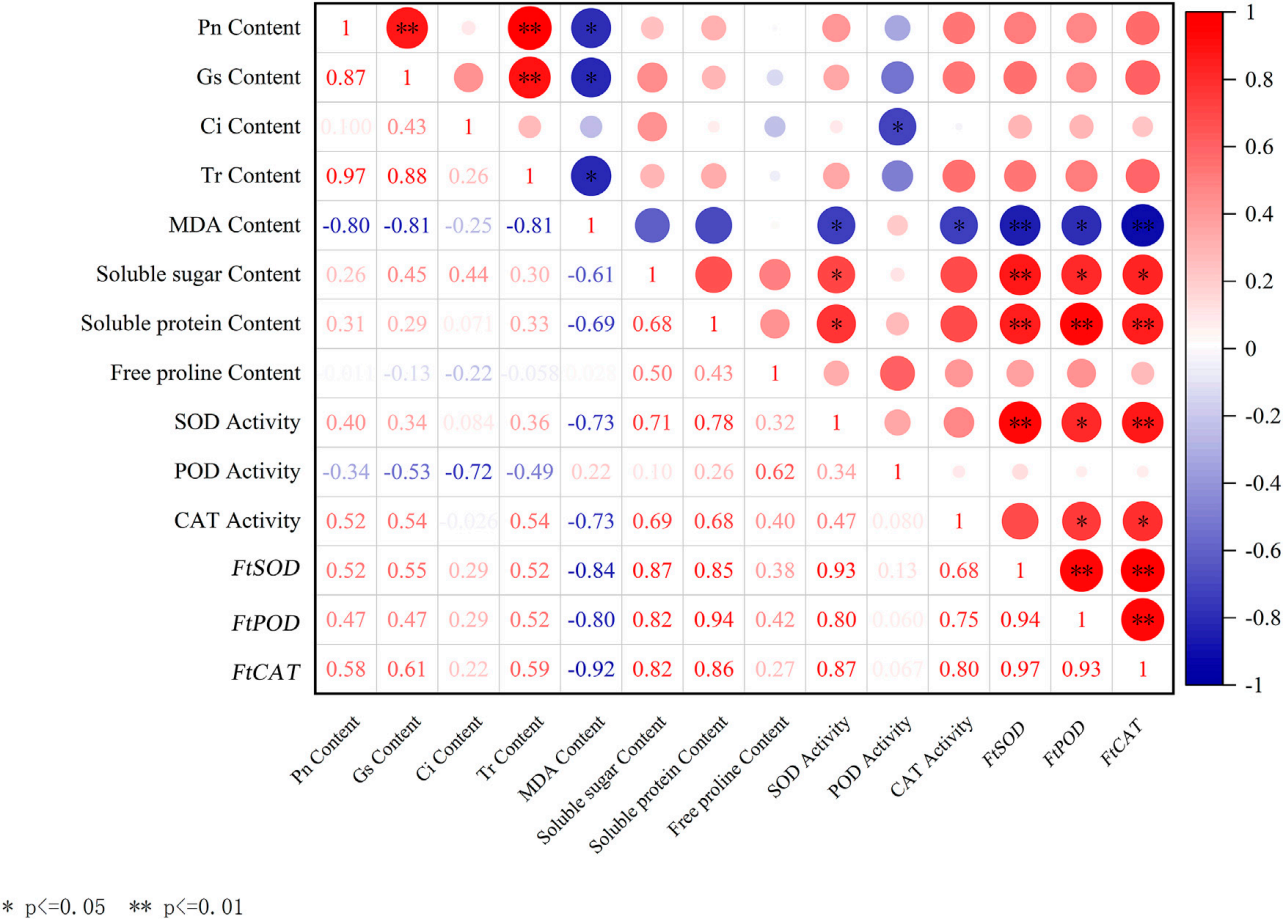
Correlation analysis between different indicators. Red in the figure indicates positive correlation, blue indicates negative correlation, and numbers indicate correlation coefficients.

In this study, the correlation analysis was performed on the photosynthetic parameters (Pn, Gs, Ci, and Tr), MDA, soluble sugar, soluble protein, and free proline contents, the activity index of the three protective enzymes, and the relative gene expression levels of the three protective enzymes (Figure 2). As shown in Figure 2, the *FtCAT* gene expression was highly positively correlated with *FtSOD* (*r* = 0.97, *P* < 0.01) and *FtPOD* (*r* = 0.93, *P* < 0.01) genes expression, but highly negatively correlated with the MDA content (*r* = 0.92, *P* < 0.01). The *FtPOD* gene expression was highly positively correlated with the soluble protein content (*r* = 0.94, *P* < 0.01) and significantly positively correlated with the soluble sugar content (*r* = 0.82, *P* < 0.05). The *FtSOD* gene expression was highly positively correlated with the soluble sugar content (*r* = 0.87, *P* < 0.01) and soluble protein content (*r* = 0.85, *P* < 0.05). The MDA content was negatively correlated with Pn (*r* = 0.80, *P* < 0.05) and Gs (*r* = 0.78, *P* < 0.05).

## 4 Discussion

Phosphorus-solubilizing fungi are rhizosphere growth-promoting strains that can convert the phosphorus in the soil, which is difficult to dissolve, into a form that plants can absorb and use. The growth condition, oxidation resistance, stress resistance, and metabolism of plants can be improved by enhancing the phosphorus absorption capacity of plants. At the same time, fungi augment the soil structure and inhibit soil impoverishment (El-Maraghy et al., 2021; Iftikhar et al., 2024). In practice, phosphorus-solubilizing fungi applied for fertilizer production can improve the fertilizer utilization rate and alleviate environmental pollution caused by the excessive use of chemical fertilizers, which is also the practical significance of this study.

In this study, the leaf photosynthetic index of *F. taipaiensis* P. Y. Li was analyzed. Photosynthesis is the main metabolic pathway through which plants produce energy (Li et al., 2023). If photosynthesis is stronger, more organic matter accumulates in plants, promoting their growth. Plant photosynthesis occurs in the leaves. The leaf area size directly determines the light absorption area of the plant. The leaf area measurement can intuitively reflect plant growth (Lopez et al., 2024). In the present study, the leaf area was significantly higher in the treatment groups than in the control group. The leaf area in the S7 group was the largest, proving that phosphorus-solubilizing fungi significantly affected the growth of *F. taipaiensis* P. Y. Li. The photosynthetic pigment content was low in the S5 group, and the leaf area was relatively small. Pn, Gs, Ci, and Tr are also closely related to plant photosynthesis (Baruah et al., 2016; Ma et al., 2021; Guo et al., 2023; Ma et al., 2023). In this study, Ci was lower in some treatment groups than in the CK group. Under normal circumstances, the increase in Ci leads to stomatal closure and weakens transpiration because of stomatal limitation, reducing Gs and Tr (Wang et al., 2017). The Ci was lower in the single-strain treatment group than in the CK group. In contrast, the Ci in the combined strain treatment group was higher than that in the CK group. Gs and Tr were significantly higher in all treatment groups than in the CK group. The combined treatment improved the stomatal restriction of plants. Therefore, the net photosynthetic rate was significantly higher in these treatment groups than in the CK group. The aforementioned results indicated that using phosphorus-solubilizing fungi reduced stomatal limitation, thereby increasing the photosynthetic rate of plants. Chlorophyll and carotenoids in leaves primarily absorb sunlight and store energy for photosynthesis (Yuan et al., 2022). The strains used also exerted significant enhancement effects on photosynthetic pigments. The combined treatment groups exerted better enhancement effects on chlorophyll a and chlorophyll b, whereas the single treatment groups were more suitable for increasing the carotenoid content. An analysis of a series of photosynthetic parameters revealed that the phosphorus-solubilizing fungi promoted plant photosynthesis by reducing stomatal restriction and increasing the contents of light-absorbing pigments, which was beneficial for the growth of *F. taipaiensis* P. Y. Li plants.

MDA is the final product of membrane lipid peroxidation during plant aging. MDA content reflects the oxidation degree of membrane lipids and thus the stress resistance of plants (Zhao et al., 2022; Shams et al., 2024). The MDA content was significantly lower in all treatment groups than in the CK group. The MDA content in the S7 group was the lowest. This proved that phosphorus-solubilizing fungi significantly inhibited the production of free radicals and reactive oxygen species, reduced the damage caused by membrane lipid peroxidation to *F. taipaiensis* P. Y. Li plants, and thus promoted the normal growth and health of plants. Soluble sugar, soluble protein, and free proline are major osmoregulatory substances and nutrients in plants (Zhang et al., 2023; Peng et al., 2024). They participate in transport and metabolism in plants, protect plant cell membranes and organelles to maintain cell structure stability, and produce functional enzymes (Haider et al., 2023). In the present study, soluble sugar and soluble protein contents were all higher in the treatment groups than in the CK group. In contrast, the free proline content was significantly lower in the S7 group than in the CK group. Based on the experimental results, we concluded that phosphorus-solubilizing fungi positively impacted the physiology and biochemistry of *F. taipaiensis* P. Y. Li. However, the combined effect of the three fungi was not good or was antagonistic. Therefore, we do not recommend their combined application. Xu et al. (2022) also reported a similar effect, which might be caused by the excessively strong competitive interaction between *A. niger* and the other two co-existing fungi. However, this conclusion only suggested that the three fungi were antagonistic to each other. Pant et al. (2024), using scanning electron microscopy, showed that *A. niger* affected the mycelia of other fungi, leading to spore rupture of co-existing fungi and inhibited growth. This might be one of the reasons why the growth-promoting effect was inhibited when the three fungi were used together. Zhang et al. (2020) isolated the secondary metabolites of *A. niger* sporangium powder and obtained compounds with significant antagonistic effects on some bacteria. However, whether they had the same effect on fungi remains unclear.

SOD accelerates the transformation of superoxide anion into H_2_O_2_ and oxygen in plants. POD and CAT eliminate H_2_O_2_. The synergistic effect of these three enzymes maintains the free radical content in plants, which is the main function of antioxidant enzymes (Wang et al., 2023; Ali et al., 2024). SOD mainly scavenges free radicals and is the primary enzyme that fights against damage induced by oxygen free radicals to plant cells. The SOD content is the main index reflecting the plant antioxidant activity. POD and CAT can assist in scavenging H_2_O_2_ and toxic substances produced and fighting against aging (Xie et al., 2022; Liu et al., 2023; Ang et al., 2023). The activities of the three enzymes were significantly higher in the treatment groups than in the CK group. The experimental results largely in alignment with those obtained through RT-qPCR. Specifically, the gene expression levels of the genes encoding these protective enzymes exhibited a highly positively correlated with the activities of these enzymes. Notably, the S5 group showed the most pronounced enhancement in the expression levels of these genes. Furthermore, the activities of the three enzymes were relatively higher in the S5 group than in the other treatment groups. These findings collectively suggest that phosphorus-solubilizing fungi promoted the reaction of antioxidant enzymes in *F. taipaiensis* P. Y. Li and enhanced the expression of genes encoding these protective enzymes.

The emerging field of rhizosphere fungal research has revealed the significant potential of rhizosphere fungi to influence plant health and development, as evidenced by numerous studies on medicinal plants. Zheng et al. (2016) selected medicinal peonies cultivated at different growth stages across five geographical areas. They used Biolog and 454 pyrosequencing technology to assess overall microbial activity and fungal diversity, and explored the relationship between the true regional characteristics of medicinal plants and rhizosphere microorganisms. They reported the diversity and regional characteristics of medicinal peony rhizosphere soil fungi. Li et al. (2023) collected and cultured fungi from the roots and rhizosphere of *Salvia miltiorrhiza*, used culturomics and high-throughput sequencing to evaluate the fungal species diversity of *S. miltiorrhiza* roots and rhizosphere, and verified the predicted cellulose in metagenomic analysis. Tang et al. (2021) examined *Fritillaria* rhizosphere microorganisms. The results showed interactions between the growth of *F. taipaiensis* P. Y. Li, soil factors, and rhizosphere microorganisms. These studies highlighted the intrinsic link between the diversity of rhizosphere fungi, the accumulation of bioactive substances, and interactions between plants. They also illustrated the important roles of rhizosphere fungi in the health and performance of medicinal plants. A key aspect of cultivation and optimization. The impact of rhizosphere fungi on medicinal plant health, biomass production, and secondary metabolite biosynthesis has received widespread attention, particularly with the application of molecular biology and omics technologies in recent years (Yang et al., 2024). However, the extent to which rhizosphere fungi can contribute to the construction of a quality evaluation system for Chinese medicinal materials remains unclear. Our study only confirmed that different rhizosphere fungi or their combinations impacted the photosynthetic characteristics, basic physiological and biochemical indicators, and protective enzyme systems of *F. taipaiensis* P. Y. Li. However, further in-depth research is required to determine how these different fungal combinations affect plant production by producing different metabolites. In summary, a comprehensive analysis of the composition of rhizosphere fungi and their interactions with host plants can provide valuable insights for improving the quality of medicinal plants and promoting the efficient utilization of biological resources (Zhu et al., 2021). It provides a reference for the ecological cultivation of Chinese medicinal materials and ensuring the production of high-quality Chinese medicinal materials in the future by regulating rhizosphere microbial communities.

## 5 Conclusion

Phosphorus-solubilizing fungi *A. tubingensis* and *A. nigerfunigatus* can promote plant photosynthesis by improving leaf stomatal conductance and increasing light-absorbing pigments. They also enhance the antioxidant enzyme activity, maintain osmotic pressure, and regulate a series of enzyme reactions in plants. Further, they help the plants in establishing a defense system against free radicals and toxic substances, enhance antioxidant activity, delay aging, and promote the growth and development of *F. taipaiensis* P. Y. Li. The effect of *A. niger* used alone was higher than that of *A. niger* used in combination with the other two fungi. The antagonistic reaction between the fungi may be a reason for this difference, but the exact cause of this antagonism remains unclear, requiring further investigation. Therefore, the development and promotion of the combination of *A. tubingensis* and *A. nigerfunigatus* during artificial cultivation can be a good technical approach to improve the quality of *F. taipaiensis* P. Y. Li, strengthen plants, enhance soil fertility, and improve the utilization rate of phosphorus. Additionally, it can reduce fertilizer pollution, improve soil conditions, and provide favorable conditions for further cultivation of *F. taipaiensis* P. Y. Li, creating a virtuous cycle in artificial cultivation.

## Data Availability

The datasets presented in this study can be found in online repositories. The names of the repository/repositories and accession number(s) can be found in the article/supplementary material.
